# Cuproptosis-related gene signatures define immune subtypes and predict prognosis in gastric cancer

**DOI:** 10.3389/fmolb.2026.1746613

**Published:** 2026-02-09

**Authors:** Zhe Wang, Yuxin Man, Bingtong Yue, Min Liu, Jiayi Zhang, Feng Wang, Dao Xin

**Affiliations:** 1 Department of Oncology, The First Affiliated Hospital of Zhengzhou University, Zhengzhou, China; 2 Tianjian Laboratory for Advanced Biomedical Sciences, Zhengzhou, China; 3 State Key Laboratory of Metabolic Dysregulation and the Prevention and Treatment of Esophageal Cancer, Zhengzhou, China; 4 Department of Medical Oncology, Sichuan Clinical Research Center for Cancer, Sichuan Cancer Hospital & Institute, Sichuan Cancer Center, University of Electronic Science and Technology of China, Chengdu, China; 5 Department of Colorectal Surgery and Oncology, Key Laboratory of Cancer Prevention and Intervention, Ministry of Education, The Second Affiliated Hospital, Zhejiang University School of Medicine, Hangzhou, Zhejiang, China; 6 Department of Oncology, Anyang District Hospital, Anyang, China

**Keywords:** cuproptosis, prognostic model, single-cell RNA sequencing (sc-RNA seq), stomach adenocarcinoma, tumor immune microenvironment

## Abstract

**Introduction:**

Despite recent improvements in diagnostic and therapeutic strategies, gastric cancer (GC) continues to be a major contributor to global cancer fatalities, resulting in suboptimal patient prognosis overall. Cuproptosis, defined as a regulated death mode initiated by intracellular copper overload, has not been comprehensively examined in the context of the tumor immune microenvironment or its prognostic relevance in stomach adenocarcinoma.

**Methods:**

Comprehensive transcriptomic analyses of TCGA and GEO cohorts were performed to identify cuproptosis-related molecular subtypes and to develop a prognostic risk model based on cuproptosis-associated genes. Correlations between the risk score and features of the TIME were thoroughly evaluated. RT-qPCR was conducted in 14 paired gastric tumor and adjacent normal tissues to validate the expression of key prognostic genes.

**Results:**

Three distinct cuproptosis-associated molecular subtypes were identified in STAD. A five-gene prognostic signature—*PEG10, RPL39L, MMP11, SYNPO2,* and *KRT17*—was established, which showed strong associations with overall survival and multiple immunological indicators, including immune cell infiltration, MSI, TMB, immune checkpoint expression, and HLA profiles. Subsequently, a nomogram provided enhanced individualized survival prediction.

**Conclusion:**

The identified cuproptosis-related gene signature is associated with the immunological heterogeneity of gastric cancer. Together, these results point to previously unrecognized links between cuproptosis and antitumor immunity, may provide insights for refining immunotherapeutic strategies in gastric cancer. Continued experimental investigation will be necessary to unravel how cuproptosis contributes to gastric cancer development and to validate its association with immune regulation.

## 1 Introduction

Gastric cancer (GC), including tumors arising at the gastroesophageal junction, remains the fourth leading contributor to cancer-associated mortality worldwide (Sung et al., 2021), with stomach adenocarcinoma (STAD) representing the dominant histological form. Most individuals are diagnosed only after metastatic spread has occurred. Although therapeutic approaches for advanced GC have evolved from traditional chemotherapy to include molecularly targeted drugs and immune-based treatments, producing incremental survival gains, the overall clinical benefit remains modest immunotherapy, which has yielded improved patient outcomes, therapeutic success is often limited (Joshi and Badgwell, 2021). The highly heterogeneous nature of GC further complicates treatment, as a considerable proportion of patients show minimal or no response to targeted agents or immunotherapy. Therefore, identifying and selecting the specific patient populations most likely to respond to these innovative treatments is paramount for enhancing overall survival rates (Seeneevassen et al., 2021).

Copper, an essential mineral nutrient, plays roles in pathways related to cellular proliferation and death. Copper was once regarded mainly as a catalytic cofactor required for metabolic enzyme activity, yet accumulating research now positions it as a dynamic signaling element and allosteric modulator within cellular systems (Gupte and Mumper, 2009). Copper-dependent control has been documented in multiple pathways, including those involving phosphodiesterase 3B and the kinases MEK1/MEK2 that influence proliferation, as well as ULK1/ULK2, which govern autophagy regulation (Ge et al., 2022). Although the exact process through which copper overload triggers cell death has not been fully clarified, a recent landmark report by Peter Tsvetkov and colleagues identified cuproptosis as a previously unrecognized cell death mechanism distinct from apoptosis, necroptosis, pyroptosis, and ferroptosis. Their findings indicated that cells with strong dependence on mitochondrial respiration are particularly vulnerable to copper-induced toxicity, suggesting a mechanistic link to the tricarboxylic acid cycle. Central to this process is protein lipoylation, regulated upstream by Ferredoxin 1 (FDX1); loss of FDX1 disrupts lipoylation of dihydrolipoamide S-acetyltransferase (DLAT) (Tsvetkov et al., 2022). The same study demonstrated that copper exposure drives DLAT oligomerization and accumulation of insoluble DLAT, ultimately generating proteotoxic stress and cell death.

**FIGURE 1:**
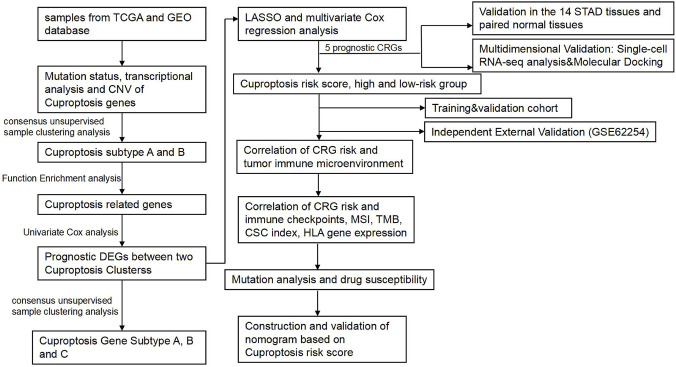
Flowchart of the present study.

Dysregulated copper metabolism has been implicated in tumor growth, angiogenesis, and immune modulation. Recently, multi-cohort studies identified distinct cuproptosis-related molecular subtypes with characteristic metabolic and immune profiles in GC, suggesting that abnormal cuproptosis activity may be associated with reprogrammed mitochondrial metabolism and alterations in the tumor microenvironment (Chong et al., 2024). Recent perspectives delineate two complementary therapeutic routes in oncology—targeting tumor-intrinsic programs and targeting the tumor’s neighbors in the microenvironment. Framing gastric cancer within these dual axes provides the rationale for examining cuproptosis together with tumor–microenvironment features in the analyses that follow (Liu and Dilger, 2025). In this work, we analyzed STAD datasets from TCGA and GEO to characterize cuproptosis-related genes (CRGs) in terms of expression, mutation patterns, and copy-number alterations. Using CRG expression signatures, samples were first separated into two cuproptosis-associated phenotypes, and subsequent gene-level refinement based on differentially expressed transcripts produced three molecular subtypes. These subgrouping results were then used to construct a prognostic framework, enabling systematic exploration of how the derived risk categories relate to features of the tumor immune microenvironment. To date, no prior study has examined the relationship between cuproptosis, immunity, and clinical outcome in STAD, and the present findings offer a basis for future efforts in biomarker refinement, therapeutic stratification, and targeted intervention in gastric cancer. A summary of the analytical workflow is presented in Figure 1.

## 2 Methods

### 2.1 Data collection

RNA sequencing data, along with corresponding clinical data, were acquired from the TCGA and GEO (GSE84433 and GSE62254) databases. The GSE62254 cohort (n = 300), also known as the ACRG cohort, was utilized as an independent external validation set to verify the generalizability of the prognostic model. For the TCGA-STAD cohort, FPKM values were transformed and normalized to TPM. We aggregated the two datasets, excluding patients lacking necessary clinical information. Consequently, 745 STAD patients were enrolled in our study. A set of 10 cuproptosis-associated genes was chosen for this study, aligning with previous publications (Supplementary Table S1).

### 2.2 Analysis of somatic mutations and copy number alterations in CRGs

Somatic mutation and copy-number datasets processed through the VarScan2 pipeline were obtained from TCGA. Mutation frequencies across the ten cuproptosis-related genes were summarized to highlight those most frequently altered, and CNV patterns were quantified in parallel. We then examined how copy-number status related to transcript abundance and mapped the chromosomal distribution of these genes.

### 2.3 Consensus clustering analysis of CRGs

ConsensusClusterPlus was applied to perform unsupervised subtype classification based on CRG expression patterns, allowing STAD cases to be partitioned into molecularly distinct groups. Subtype selection was guided by stability criteria, including a smoothly rising cumulative distribution function and avoidance of highly uneven sample allocation across clusters.

### 2.4 Assessment of cuproptosis subtypes in relation to clinicopathology and prognosis

To determine the clinical significance of the molecular subgroups, we compared them against pathological features, CRG expression patterns, and patient outcomes. The variables examined included age, sex, tumor stage, and histological grade. Differences in overall survival among the subtypes were then evaluated using K-M analysis.

### 2.5 Correlating molecular subtypes with immune cell infiltration and function

Immune and stromal characteristics for each STAD case were first inferred using the ESTIMATE scoring system. Next, we estimated relative infiltration levels across multiple immune cell populations via ssGSEA. Enrichment profiles were then used to evaluate multiple immune-related functional programs, including antigen-presentation mechanisms, checkpoint regulation, HLA activity, T-cell stimulatory or suppressive signaling, and IFN-γ–associated pathways.

### 2.6 Identification and functional profiling of differentially expressed genes (DEGs)

Transcriptomic differences between the cuproptosis-related groups were first screened using limma in R, applying *p* < 0.05 and a 1.5-fold expression threshold. To explore pathway-level heterogeneity, GSVA was performed with KEGG-based gene sets (c2.cp.kegg.v7.4.symbols.gmt) sourced from MSigDB. The resulting gene list was then subjected to enrichment analysis using clusterProfiler and visualized with enrichplot to determine biologically relevant signaling patterns.

### 2.7 Derivation of a cuproptosis-related prognostic risk score

The cuproptosis-related prognostic risk score (CRG score) was formulated to numerically represent the cuproptosis pattern in each STAD specimen. Initial selection of DEGs linked to OS was performed via univariate Cox regression analysis. These prognostic DEGs were then used for unsupervised clustering, partitioning all patients into distinct cuproptosis gene clusters (A, B, C). All STAD cases (n = 745) from the integrated TCGA and GEO datasets were randomly split (1:1 ratio) into a training cohort (n = 373) and a validation cohort (n = 372). The training dataset served as the foundation for constructing the CRG score. Next, Lasso regression (R package “glmnet”) was employed, which was ultimately followed by multivariate Cox regression analysis to pinpoint the final set of candidates prognostic cuproptosis genes. Patients were subsequently divided into high- and low-risk strata using the median CRG score as the cutoff threshold. A correlation plot illustrated the relationship between OS status and the CRG score. To confirm the robustness and external applicability of the signature, the same risk scoring formula was applied to the independent validation cohort (GSE62254, n = 300). Patients in this external cohort were similarly stratified into high- and low-risk groups based on the median risk score calculated within that specific cohort.

### 2.8 Immune landscape, MSI, CSC, TMB, and HLA expression in risk groups

Using the CRG-based risk measure, we assessed its linkage with broad immune cell composition via CIBERSORT and compared major stromal–immune features across risk categories with ESTIMATE. We next examined how individual prognostic CRGs aligned with immune infiltration patterns and evaluated the score in the context of immunotherapy-related indicators, including checkpoint expression, genomic instability, and tumor mutation characteristics. Finally, mutational differences between high- and low-risk tumors in the TCGA cohort were profiled using maftools.

### 2.9 Drug sensibility analysis

To explore the potential of the CRG score in guiding treatment selection, we applied the pRRophetic framework to model drug sensitivity. Predicted IC50 values for commonly used chemotherapeutic and targeted agents were then contrasted between the two stratified risk categories.

### 2.10 Development and validation of a CRG-based nomogram

Independent survival determinants were first screened using Cox regression, after which a nomogram combining the CRG score with key clinical features was generated to estimate long-term survival. Model performance was examined using calibration assessments and its potential clinical usefulness was evaluated through decision curve analysis (DCA).

### 2.11 Clinical samples and quantitative real-time polymerase chain reaction (RT-qPCR)

Tissue samples consisted of 14 primary gastric carcinomas and their paired adjacent normal mucosa, obtained from patients undergoing resection at the First Affiliated Hospital of Zhengzhou University. Ethical clearance was granted by the institutional review committee (No. 2023-KY-0951-002). RNA was extracted using Trizol (Takara, Beijing, China), after which complementary DNA was produced with the PrimeScript RT kit equipped with a gDNA removal step. qRT-PCR amplification was then performed using SYBR Green reagents (Cowin Biosciences, Jiangsu, China) along with primers sourced from Sangon Biotech (Shanghai, China). GAPDH expression served as the internal reference for normalization.

### 2.12 Single-cell RNA sequencing analysis

The single-cell RNA-seq dataset (GSE163558) was acquired from the Gene Expression Omnibus (GEO) database. Raw data processing, quality control, and downstream analyses were performed using the Seurat package (version 5.0.1) within the R statistical environment (version 4.3.2). Strict quality control measures were implemented to filter out low-quality cells and potential doublets. Following data normalization and the identification of highly variable features, Principal Component Analysis (PCA) was applied for dimensionality reduction. Cluster visualization was subsequently achieved using the Uniform Manifold Approximation and Projection (UMAP) algorithm. Cell types were annotated based on the expression profiles of canonical marker genes. Finally, feature plots and violin plots were generated to visualize the specific expression patterns of risk genes across the identified cell clusters.

### 2.13 Molecular docking analysis

To investigate the potential protein-protein interaction between the core risk gene KRT17 and the cuproptosis regulator FDX1, molecular docking simulations were conducted using the HDOCK server (http://hdock.phys.hust.edu.cn/). This platform employs a hybrid algorithm combining template-based modeling and *ab initio* free docking. The protein structures were docked to calculate binding energies and docking scores. The model exhibiting the highest confidence score and the lowest binding energy was selected as the optimal binding conformation. The 3D visualization and structural analysis of the docking results were performed using PyMOL software (version 2.5.4; Schrödinger, Inc.).

### 2.14 Statistical analysis

All analyses were carried out in R (version 4.1.1). Group comparisons followed standard statistical procedures, with Student’s t-tests or Wilcoxon tests applied to two-group contrasts and ANOVA used when evaluating multiple groups. A *p*-value threshold of 0.05 was considered statistically significant.

## 3 Results

### 3.1 Genomic alterations of cuproptosis-related genes in STAD

**FIGURE 2:**
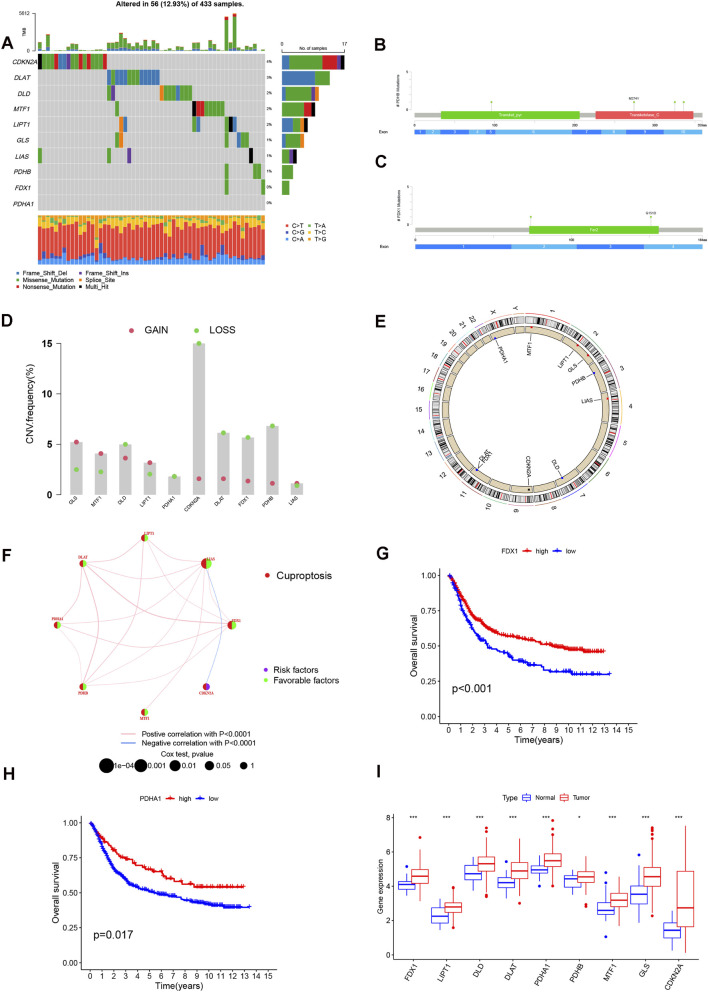
Genetic and transcriptional analysis of cuproptosis-related genes (CRGs) in STAD. **(A)** Mutation frequencies of 10 CRGs in STAD patients from TCGA. **(B,C)** Mutational analysis of PDHB and FDX1 according to the cBioPortal database. **(D)** Copy number variation (CNV) of CRGs. **(E)** Locations of CRGs CNV on 23 chromosomes. **(F)** Interactions among CRGs in STAD, where blue and pink lines indicate negative and positive correlations, respectively. **(G,H)** Kaplan-Meier survival analysis based on expression levels of FDX1 and PDHA1. **(I)** Differential expression of CRGs between normal and STAD samples.

Ten cuproptosis-related genes were examined in the STAD cohort. Somatic alterations occurred in roughly 13% of cases, with CDKN2A showing the highest mutation burden, followed by DLAT and several others at lower frequencies, while no mutations were detected in FDX1 or PDHA1 (Figure 2A). Representative mutation sites retrieved from cBioPortal included substitutions in PDHB and FDX1 (Figures 2B,C). Supplementary Figure S1 presents the common mutation site of other cuproptosis genes. Copy-number profiling revealed distinct alteration patterns, characterized by gains in GLS, MTF1, and LIPT1, whereas losses were observed in CDKN2A, DLAT, FDX1, PDHB, and DLD (Figure 2D). Most genes demonstrated significantly increased expression in tumor tissue compared with normal controls (Figure 2E). An analysis of copy number variations (CNVs) revealed that cuproptosis-associated genes exhibiting CNV gain, specifically GLS, MTF1, and LIPT1, showed a marked upregulation in their expression levels within STAD specimens. This finding suggests a potential mechanism where CNV influences the transcriptional activity of cuproptosis genes. Conversely, genes characterized by CNV deletion nonetheless presented with heightened messenger RNA (mRNA) abundance. Moreover, certain genes, such as LIAS, demonstrated no change in expression when contrasting tumor versus normal samples, despite frequent CNV gains or losses. This collective evidence implies that the control of cuproptosis gene expression is not solely dictated by CNV status.

### 3.2 Stratification of STAD patients by cuproptosis expression patterns

Initial analysis employed univariate Cox regression to establish the prognostic relevance of the entire panel of ten cuproptosis genes in STAD patients (Supplementary Table S2). Drawing upon these regression findings, a prognostic network for cuproptosis was created, which mapped the overall pattern, reciprocal connections, and predictive capability of each cuproptosis gene within STAD (Figure 2F). Among the analyzed genes, five cuproptosis regulators-FDX1, LIAS, LIPT1, DLAT, and PDHA1-showed significant associations with overall survival. K-M analysis demonstrated that higher expression of these genes corresponded with improved survival outcomes in STAD patients (Figures 2G,H; Supplementary Figure S2). In addition, most cuproptosis genes displayed notable expression differences between tumor and adjacent normal tissue (Figure 2I).

**FIGURE 3:**
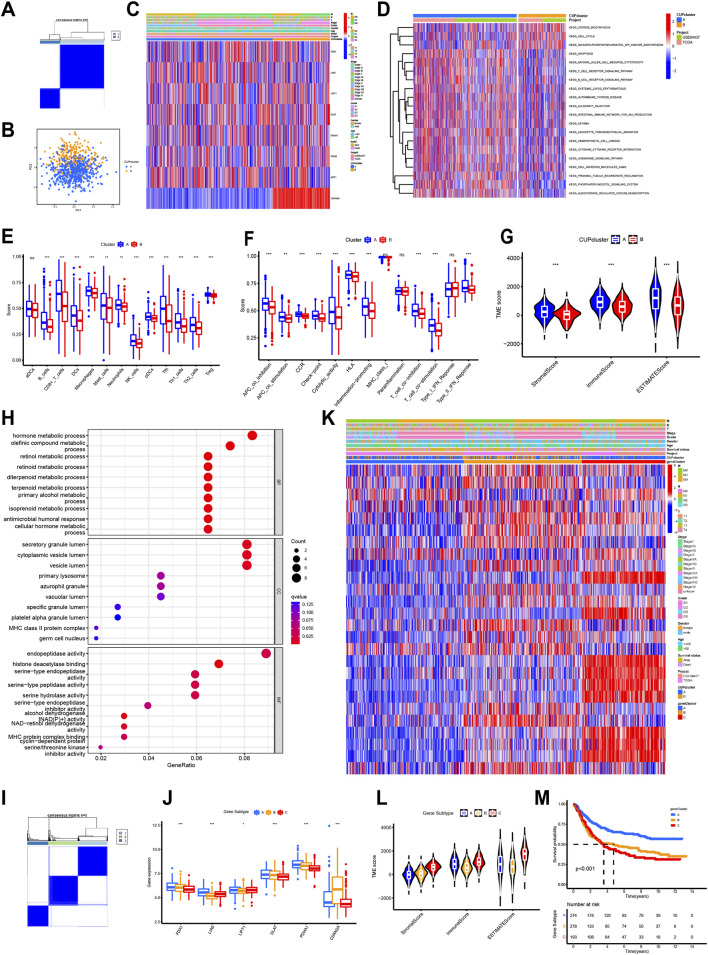
Cuproptosis subtypes and gene clusters in STAD. **(A)** Consensus matrix heatmap identifying 2 clusters (k = 2). Dark blue indicates low consensus (0) and white indicates high consensus (1). **(B)** PCA analysis showing a significant transcriptional distinction between Cluster A (red) and Cluster B (blue). **(C)** Differences in clinicopathological factors and CRG expression between the two clusters. Red represents high expression/presence, and blue represents low expression/absence. **(D)** GSVA analysis of biological pathways; red bars indicate activated pathways in Cluster A, and blue bars indicate activated pathways in Cluster B. **(E)** Immune cells infiltrate between different cuproptosis clusters. **(F)** Immune functions between different cuproptosis clusters. **(G)** Correlation of cuproptosis clusters with immune/stromal scores. **(H)** GO analysis of DEGs between two cuproptosis clusters. **(I)** Consensus matrix heatmap identifying 3 gene subtypes (k = 3). **(J)** Differences in CRG expression between three gene subtypes. **(K)** Differences in clinicopathological factors and CRGs expression between three gene subtypes. **(L)** Correlation of gene subtypes with immune/stromal scores. **(M)** Kaplan-Meier survival analysis of the three gene subtypes.

Using consensus clustering of CRG expression profiles, the cohort was partitioned into two stable molecular groups (k = 2), designated Cluster A and Cluster B (Figure 3A; Supplementary Figure S3; Supplementary Table S3). This selected partition parameter ensured a comparative balance in the patient counts across the two resultant subgroups. Principal Component Analysis (PCA) demonstrated that the cuproptosis transcriptional landscape displayed a statistically significant distinction between Cluster A and Cluster B (Figure 3B). Despite this genetic separation, no significant differences in OS were noticed between the two groups (*p* = 0.487; Supplementary Figure S4). However, the subtypes demonstrated clear distinctions in CRG expression patterns and baseline clinical features (Figure 3C).

### 3.3 Functional divergence and tumor microenvironment profiles across cuproptosis subtypes

GSVA was used to characterize the biological divergence between the two cuproptosis-associated subgroups, and the resulting profiles indicated that Subtype B was predominantly linked to pathways governing cell-cycle progression and steroid biosynthesis, whereas Cluster A demonstrated a markedly different pattern defined by activation of apoptosis-related signalling and multiple immune pathways, including NK-cell cytotoxicity, adaptive receptor signalling, leukocyte migration, and chemokine networks (Figure 3D; Supplementary Table S4).

When ssGSEA was applied to quantify immune infiltration, a clear stratification emerged: most immune cell types showed substantially higher infiltration in Cluster A, matching the immune-enriched functional signature detected in GSVA (Figure 3E). This pattern was further reflected in immune activity scoring, where Cluster A consistently exhibited stronger enrichment across diverse immune functional categories, such as antigen presentation, immune checkpoint activity, cytolytic capacity, CCR signalling, HLA processes, inflammatory signalling, and type II IFN-γ responses (Figure 3F). To further contextualize these findings within the tumor microenvironment, ESTIMATE scoring was performed, and the results showed that Cluster A carried both elevated stromal and immune scores, suggesting a microenvironment characterized by higher stromal presence and heightened immune engagement compared with Cluster B (Figure 3G).

### 3.4 Gene-based cuproptosis classification derived from survival-associated DEGs

Differential expression analysis identified 103 cuproptosis-related differentially expressed genes (DEGs) distinguishing Clusters A and B, a process utilizing the R package “limma” (Supplementary Table S5). Functional annotation followed, beginning with GO analysis. This revealed significant enrichment of the DEGs in biological processes linked to the metabolism of intracellular components (Figure 3H).

Subsequently, a univariate Cox regression analysis was performed, yielding 26 prognostic DEGs that correlated with OS in STAD (*p* < 0.05, Supplementary Table S6). These prognostic DEGs then served as the basis for a further consensus clustering step aimed at uncovering underlying regulatory patterns. This refined analysis categorized STAD patients into three cuproptosis gene subtypes (*k* = 3, Figure 3I; Supplementary Figure S5; Supplementary Table S7). Expression levels of 6 key CRGs showed significant variations across these three resulting gene subtypes (Figure 3J). Across the three gene-defined groups, clinical characteristics were not evenly distributed, as illustrated in Figure 3K. When evaluating the tumor microenvironment, ESTIMATE scores indicated that Subtype C stood out with the highest stromal signal and a comparatively strong immune component (Figure 3L). Survival patterns further separated the subtypes, with Kaplan-Meier analysis showing that patients in Subtype A experienced the most favorable outcomes, while those in Subtype C had the shortest survival time (*p* < 0.001, Figure 3M).

### 3.5 Development and assessment of a cuproptosis-driven prognostic scoring system

**FIGURE 4:**
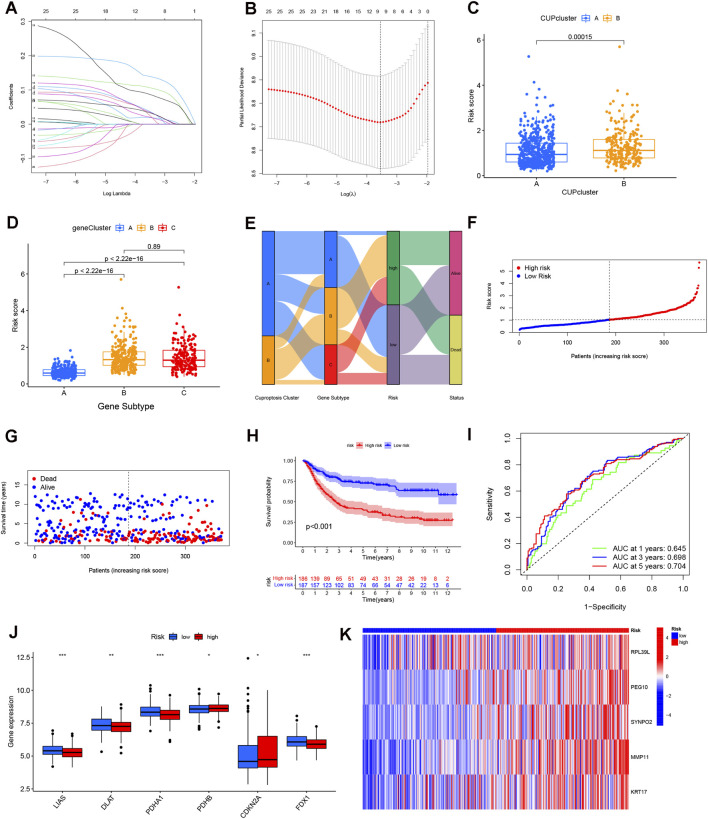
Construction of the cuproptosis gene risk score for STAD patients. **(A)** LASSO coefficient profiles of the prognostic genes. **(B)** Partial likelihood deviance plot for LASSO regression parameter selection. **(C,D)** CRG risk score distribution across cuproptosis clusters and gene subtypes. **(E)** Sankey diagram visualizing the flow of patient assignment from Clusters to Gene Subtypes, Risk Groups, and Survival Status. **(F,G)** Ranked dot plot (risk score) and scatter plot (survival status) showing that higher risk scores correlate with increased mortality (red dots). **(H)** Kaplan-Meier curve showing significantly poorer OS in the High-Risk group (red line) compared to the Low-Risk group (blue line). **(I)** Time-dependent ROC curves for predicting 1-, 3-, and 5-year OS. **(J,K)** Heatmaps showing the differential expression of the 5 prognostic model genes.

The complete collection of STAD patient data from the TCGA and GEO cohorts was combined and subsequently partitioned into a training set and a validation set at an equal ratio (Figures 4A,B). This process concluded with the identification of five prognostic cuproptosis-related genes (CRGs) identified as contributing to high risk: ribosomal protein L39 like (RPL39L), paternally expressed gene 10 (PEG10), Synaptopodin 2 (SYNPO2), matrix metallopeptidase 11 (MMP11), and Keratin 17 (KRT17) (Supplementary Table S8). The CRG risk score was formulated using the coefficients derived from the multivariate Cox regression as: CRG risk score = (0.1098 * expression of RPL39L) + (0.1460 * expression of PEG10) + (0.1657* expression of SYNPO2) + (0.1701* expression of MMP11) + (0.0598* expression of KRT17). All STAD patients were then stratified into two cohorts, designated high-risk and low-risk, based on the median value of this calculated CRG risk score.

Analysis showed that the CRG risk score varied significantly across the two initial cuproptosis clusters and the three refined gene subtypes (Figures 4C,D). Gene subtype A exhibited the lowest mean CRG score. Notably, there was no meaningful statistical difference detected between gene subtypes B and C, a finding consistent with parallel K-M results. A Sankey plot was constructed to display how the two cuproptosis clusters, the three gene-defined subtypes, the calculated risk score, and patient survival status intersect across the cohort (Figure 4E).

Distribution analysis plots illustrated an inverse relationship, showing that OS survival time in STAD decreased corresponding to an elevation in the CRG risk score (Figures 4F,G). Similar correlation patterns were established when examining the entire patient cohort and the designated validation set (Supplementary Figure S6). Survival analysis demonstrated a clear separation between groups, with the low-risk population showing markedly longer OS compared with the high-risk group (*p* < 0.001, Figure 4H). ROC analysis further supported the model’s prognostic utility, with AUC values of 0.645, 0.698, and 0.704 for predicting 1-, 3-, and 5-year survival, respectively (Figure 4I), and similar performance was observed in the full and external validation sets (Supplementary Figure S7).

To further substantiate the robustness and clinical generalizability of the 5-gene signature, we employed the GSE62254 dataset ((also known as the ACRG cohort, n = 300) as a large-scale independent external validation cohort. We calculated the risk score for each patient in the ACRG cohort using the same formula constructed in the training set and stratified patients into high- and low-risk groups based on the median risk score. Consistent with our internal findings, Kaplan-Meier analysis revealed that patients in the high-risk group of the ACRG cohort exhibited significantly worse overall survival compared to those in the low-risk group (p < 0.0001, Supplementary Figure S8). These results strongly confirm that our cuproptosis-related prognostic model maintains high stability and predictive power across different populations and sequencing platforms.

Several cuproptosis-related genes showed distinct expression differences between high- and low-risk groups (Figure 4J). A heatmap of the training set demonstrated that all five prognostic markers were expressed at higher levels in the high-risk category (Figure 4K). Clinical comparison based on risk stratification showed that adverse features such as grade 3 tumors and T3/T4 staging occurred more frequently in the high-risk cohort (Table 1).

**TABLE 1:** Patient characteristics based on risk score.^a^

Characteristic	Low risk	High risk	p-value^b^
	N = 144	N = 134	
Age, years			0.28
<65	59 (41%)	64 (48%)	
≥65	85 (59%)	70 (52%)	
Gender			0.62
Male	90 (62%)	79 (59%)	
Female	54 (38%)	55 (41%)	
Grade			0.035
1–2	62 (43%)	41 (31%)	
3	82 (57%)	93 (69%)	
Stage			0.083
1	27 (19%)	11 (8.2%)	
2	45 (31%)	47 (35%)	
3	59 (41%)	61 (46%)	
4	13 (9.0%)	15 (11%)	
T			<0.001
1	14 (9.7%)	0 (0%)	
2	31 (22%)	28 (21%)	
3	68 (47%)	69 (51%)	
4	31 (22%)	37 (28%)	
N			0.88
0	47 (33%)	39 (29%)	
1	39 (27%)	35 (26%)	
2	30 (21%)	32 (24%)	
3	28 (19%)	28 (21%)	
M			0.8
0	136 (94.4)	125 (93.3%)	
1	8 (5.6%)	9 (6.7%)	
RPL39L			<0.001
Median (IQR)	6.33 (5.23, 7.27)	7.28 (6.54, 7.89)	
Range	3.68, 9.16	3.97, 9.43	
PEG10			<0.001
Median (IQR)	3.41 (3.17, 4.17)	5.28 (4.32, 6.46)	
Range	2.90, 6.69	3.14, 10.56	
SYNPO2			<0.001
Median (IQR)	4.97 (4.35, 5.83)	6.65 (5.54, 8.60)	
Range	3.43, 8.44	3.78, 12.39	
MMP11			<0.001
Median (IQR)	6.68 (5.71, 7.56)	8.56 (7.38, 9.68)	
Range	3.59, 10.48	4.85, 12.57	
KRT17			<0.001
Median (IQR)	6.36 (4.71, 8.11)	7.94 (6.20, 9.70)	
Range	3.32, 13.17	3.40, 13.69	

^a^ Excluding patients with missing information of age, follow-up, pathological grade, and AJCC, stage.

^b^ Fisher’s exact test; Welch Two Sample t-test.

### 3.6 Experimental validation of the five cuproptosis markers included in the prognostic model

**FIGURE 5:**
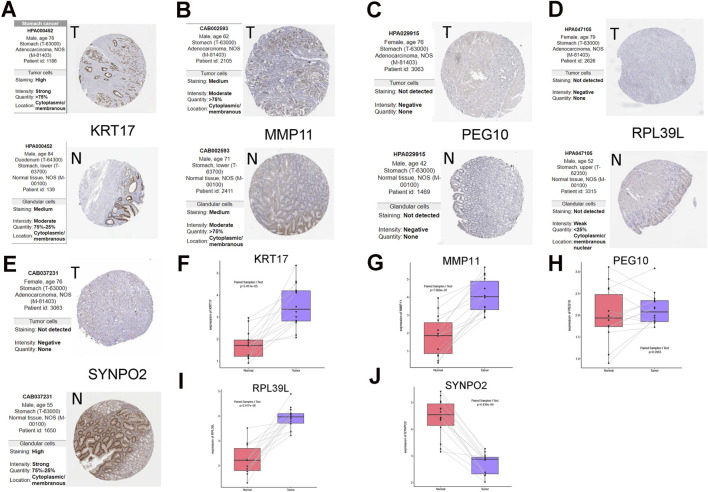
Expression validation of the 5 prognostic cuproptosis genes. **(A–E)** Protein expression levels of the model genes according to the Human Protein Atlas (HPA) database. The specific dataset and image source are clearly indicated in the figure legend. **(F–J)** Validation of mRNA transcription levels via clinical samples.

Protein expression data from HPA repository indicated differential protein abundance in STAD versus normal tissue for some of the signature genes. KRT17 protein levels were higher in STAD, implying its possible function as a risk factor. In contrast, RPL39L and SYNPO2 displayed reduced expression in tumor samples, suggesting they may serve protective roles. Notably, no significant differences in the protein expression of MMP11 and PEG10 were observed (Figures 5A–E).

To verify these observations at the transcriptional level, RT-qPCR was conducted using fourteen matched pairs of STAD and adjacent normal tissues. The transcript-level validation (Figures 5F–J) showed that KRT17, MMP11, and RPL39L were substantially overexpressed in the tumor samples, while SYNPO2 expression was diminished. Transcript levels of PEG10 showed no significant difference. Comprehensive RT-qPCR data and associated primer sequences are provided in Supplementary Tables S9, S10.

### 3.7 Single-cell resolution analysis and molecular docking reveal the tumor-intrinsic mechanism of KRT17

To further elucidate the cellular source and potential molecular mechanism of the risk signature, we performed single-cell RNA-seq and molecular docking analyses. Among the five genes included in the prognostic model, KRT17 was identified as the most critical risk component with the strongest correlation with poor survival, thus serving as the representative target for mechanistic exploration.

**FIGURE 6:**
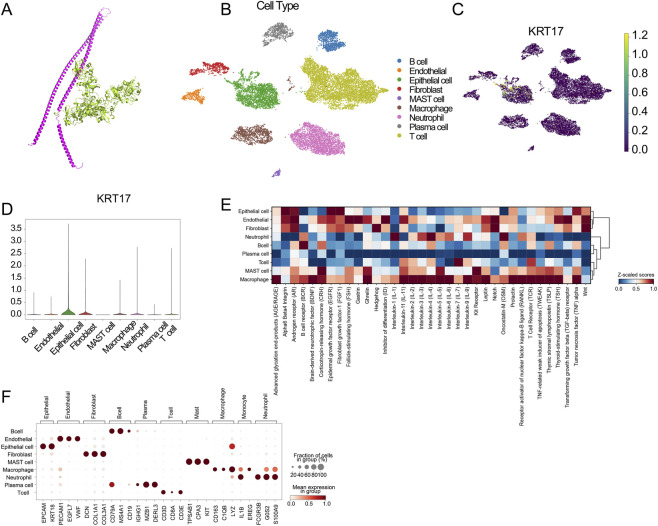
Mechanistic and single-cell resolution analysis of the core risk gene KRT17. **(A)** Molecular docking simulation revealing the physical interaction between KRT17 and the cuproptosis regulator FDX1 (Binding Score: −204.18). **(B)** UMAP visualization of major cell types in the gastric cancer single-cell cohort (GSE163558). **(C)** Feature plot demonstrating the predominant expression of KRT17 in malignant epithelial cells. **(D)** Violin plot confirming the enrichment of KRT17 expression in epithelial cells. **(E)** Heatmap showing pathway activity differences across distinct cell types. **(F)** Dot plot validating cell type annotations using canonical marker genes.

First, we explored the potential interaction between KRT17 and the cuproptosis machinery. Molecular docking simulation visualized a stable complex formed between the KRT17 protein and the core cuproptosis regulator FDX1, with a remarkably low binding energy of −204.18 kcal/mol (Figure 6A). To verify the robustness of this interaction, we screened the top 100 docking models, which consistently demonstrated high thermodynamic stability (Supplementary Table S11). This strong thermodynamic affinity suggests that KRT17 may physically sequestrate or modulate FDX1, thereby interfering with the copper-induced cell death process.

Next, to validate the cellular origin of KRT17, we analyzed the single-cell transcriptomic dataset GSE163558. Prior to downstream analysis, rigorous quality control was performed to filter out low-quality cells and doublets, ensuring the reliability of the dataset (Supplementary Figure S9). Using the UMAP algorithm, we successfully deconvoluted the tumor microenvironment into nine distinct cell clusters, including B cells, T cells, macrophages, endothelial cells, fibroblasts, and malignant epithelial cells (Figure 6B). Identity validation using canonical markers confirmed the accuracy of cell annotation (Figure 6F).

Visualizing the expression distribution of KRT17 revealed a striking pattern: unlike immune markers, KRT17 was predominantly enriched in the malignant epithelial cell cluster, with negligible expression in immune lineages such as T cells or macrophages (Figures 6C,D). Furthermore, pathway activity analysis (GSVA) highlighted significant heterogeneity in signaling pathway activation across these cell types, suggesting that KRT17-high tumor cells may harbor distinct oncogenic features (Figure 6E).

Collectively, these results provide robust evidence that the high-risk signature is driven by tumor-intrinsic alterations (specifically KRT17 overexpression) rather than primary immune cell dysfunction, which may subsequently influence the immune microenvironment.

### 3.8 Underlying TIME landscape in the cuproptosis risk model

**FIGURE 7:**
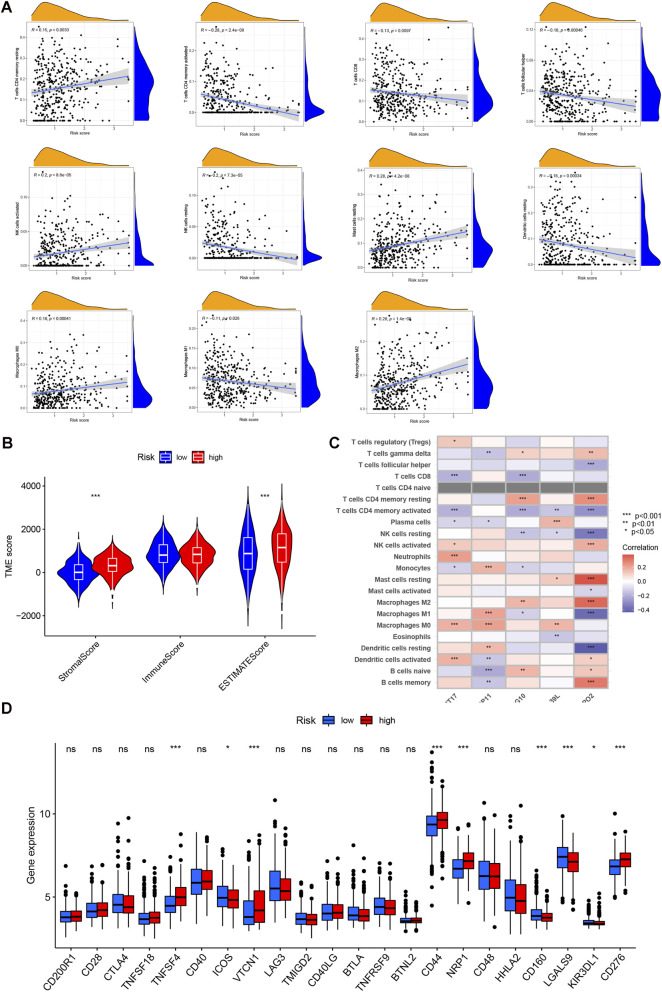
Association of TIME with the cuproptosis risk score. **(A)** Correlation of infiltrating immune cells with CRG risk score. Red bubbles indicate positive correlation, and blue bubbles indicate negative correlation. **(B)** Correlation of CRG risk score with ESTIMATE Immune/Stromal scores. **(C)** Correlation heatmap of immune cells with the 5 individual model genes. **(D)** Differential expression of immune checkpoint genes between Low-Risk (blue) and High-Risk (red) groups.

Using CIBERSORT to quantify immune infiltration, a clear relationship emerged between the CRG-based risk signature and multiple immune cell populations (Figure 7A; Supplementary Table S12). Using the ESTIMATE algorithm, we observed that stromal scores increased in parallel with higher CRG risk classifications in STAD, while immune scores showed no meaningful separation between the high- and low-risk groups (Figure 7B; Supplementary Table S13).

To extend this analysis to the immune landscape, expression of the five prognostic cuproptosis genes was systematically correlated with inferred abundances of 22 distinct immune cell populations (Figure 7C). Critically, the SYNPO2 gene correlated positively with resting memory CD4^+^ T cells and M2 macrophages, yet negatively correlated with M1 macrophages. This observation suggests that SYNPO2 may be closely involved in the interaction between cuproptosis and the TIME. Further assessment of the CRG prognostic risk score’s relationship with immune checkpoint molecules was performed (Figure 7D). This analysis identified nine immune checkpoints exhibiting differential expression between the distinct cuproptosis risk groups: tumor necrosis factor superfamily member 4, inducible T-cell CO-Stimulator, CD44, Neuropilin-1, CD276, KIR3DL1, LGALS9, and CD160.

### 3.9 Correlations of CRG risk score with MSI, CSC index, HLA gene expression, and TMB score

**FIGURE 8:**
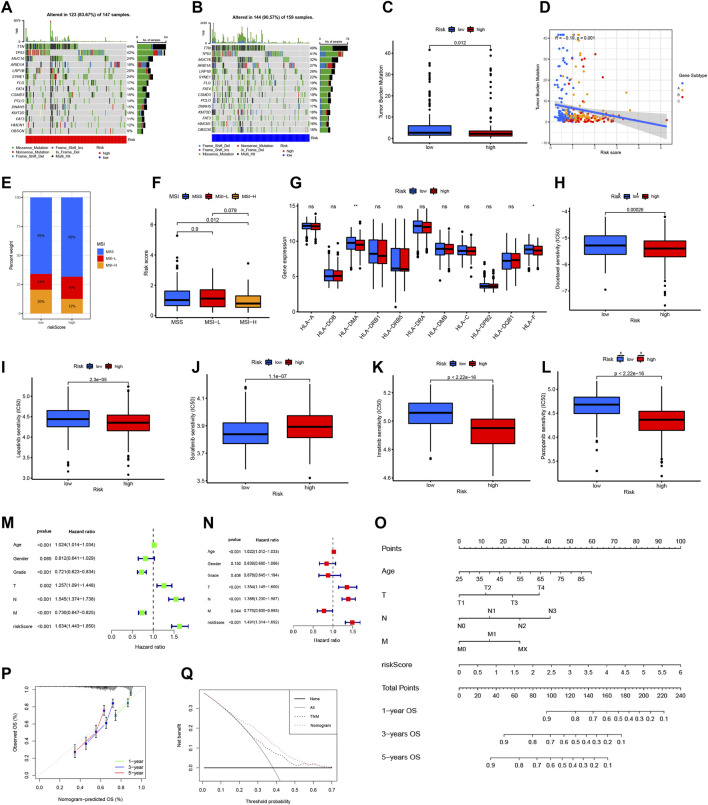
Comprehensive analysis of the cuproptosis risk score and construction of a prognostic nomogram in STAD. **(A,B)** Comparison of somatic mutation landscapes (waterfall plots) between High- and Low-Risk groups. **(C–F)** Correlations of CRG risk score with Tumor Mutational Burden (TMB), Cancer Stem Cell (CSC) index, and Microsatellite Instability (MSI) status. **(G)** Differential expression of HLA genes. **(H–L)** Predicted drug sensitivity (IC50) for chemotherapy and targeted agents; lower IC50 indicates higher sensitivity. **(M,N)** Univariate and multivariate Cox regression identifying independent prognostic factors. **(O)** Nomogram for predicting 1-, 3-, and 5-year OS probabilities. **(P)** Calibration curves demonstrating the agreement between predicted and observed survival. **(Q)** Decision Curve Analysis (DCA) showing the net clinical benefit of the nomogram compared to TNM staging.

To explore how the CRG-based risk model relates to immunotherapy-relevant features, we compared somatic alterations and biomarker patterns between the two risk groups. The low-risk subgroup demonstrated a higher mutation rate (90.57%) than the high-risk subgroup (83.67%) (Figures 8A,B), with TTN and TP53 being the most common mutations. TMB was substantially reduced in the high-risk group and showed a negative association with the cuproptosis signature (Figure 8C; Supplementary Figure S10). The CRG score also correlated inversely with the CSC index, indicating that higher-risk tumors displayed fewer stem-like features and a more differentiated phenotype (Figure 8D). MSI patterns further supported this trend: MSI-H occurred more frequently in the low-risk group, and MSI-H cases carried lower risk scores than MSI-L or MSS tumors (Figures 8E,F). Analysis of antigen-presentation–related genes showed reduced expression of HLA-DMA and HLA-F in high-risk tumors compared with low-risk samples (Figure 8G).

Predicted drug response profiling revealed treatment-related divergence between subgroups. High-risk patients appeared more responsive to docetaxel, lapatinib, pazopanib, and imatinib, as reflected by lower estimated IC50 values, whereas sorafenib sensitivity was greater in the low-risk group (Figures 8H–L). Collectively, these data suggest that the CRG-based score may provide value for predicting immune features and informing therapy selection in STAD.

### 3.10 Construction and validation of nomogram based on CRG risk score

Survival determinants were explored by first fitting univariate and then multivariate Cox proportional hazards models, through which age, TNM stage, and the cuproptosis-based risk index emerged as variables with independent prognostic weight in STAD (Figures 8M,N). These factors were subsequently incorporated into a graphical prediction tool, allowing clinicians to read off overall survival probabilities at early, intermediate, and later follow-up intervals corresponding to approximately 1, 3, and 5 years (Figure 8O). The resulting nomogram yielded a C-index of 0.746, consistent with robust discrimination. Agreement between estimated and actual outcomes was further supported by well-aligned calibration curves across all evaluated time windows (Figure 8P). In decision curve analysis, this integrated model provided a higher net clinical benefit than use of the TNM staging system alone (Figure 8Q).

## 4 Discussion

Cuproptosis has been recently characterized as a distinct form of regulated cell death triggered by intracellular copper overload and the aggregation of lipoylated TCA cycle proteins, particularly DLAT (Wang et al., 2022). While dysregulated copper metabolism is known to facilitate tumor progression, angiogenesis, and metastasis (Blockhuys et al., 2017; Shanbhag et al., 2019), the specific involvement of cuproptosis in shaping the tumor immune microenvironment TIME and its prognostic value in stomach adenocarcinoma STAD remain largely unexplored. In this study, we systematically integrated genomic and transcriptomic analyses to identify distinct cuproptosis-associated molecular subtypes, construct a robust prognostic risk signature, and comprehensively evaluate its association with immune infiltration landscapes and therapeutic responses in gastric cancer.

Currently, prognostic reporting on the predictive significance of cuproptosis-related genes within the context of tumor prognosis and the tumor immune environment remains absent. To characterize cuproptosis involvement in STAD, we analyzed CRG genomic and transcriptional patterns. Initial CRG-based clustering separated patients into two groups with distinct pathway features but no survival difference: one enriched in steroid metabolism and the other in immune-related signaling. From the resulting transcriptional differences, 26 survival-associated genes were identified and used to generate three refined molecular subtypes, which showed clear prognostic separation and distinct CRG expression patterns. These results indicate that CRG-related transcriptional signatures may serve as prognostic markers and may help predict immunotherapy response in STAD.

A validated CRG-based scoring system enabled separation of patients into high- and low-risk categories, with the low-risk group showing superior survival. Immune profiling revealed opposite infiltration trends across multiple immune cell types, including higher resting memory CD4^+^ T cells and activated NK cells but reduced activated memory CD4^+^ T cells and CD8^+^ T cells in the high-risk group. Predicted drug response patterns further indicated that cuproptosis-related signatures may assist in therapy selection. These findings supported the construction of a streamlined nomogram based on cuproptosis genes for survival prediction in STAD. Although the CRG risk score’s AUC values are modest, time dependent ROC analysis shows that these values rise from 0.645 at 1 year to 0.704 at 5 years. This upward trend implies that the subset of patients who truly benefit in the long term has not yet been clearly defined and that further refinement and stratification are needed.

To further support the biological plausibility of the prognostic model, we reviewed the reported functions of the five CRGs included in the signature. RPL39L and PEG10 have been identified as oncogenic drivers that promote proliferation, invasion, and poor prognosis across several tumor types (Wong et al., 2014). SYNPO2 generally acts as a tumor suppressor by regulating autophagy, though it may exert pro-metastatic effects in specific contexts (Zheng and Song, 2023). MMP11 contributes to extracellular-matrix remodeling, epithelial–mesenchymal transition, and immune modulation, facilitating tumor invasion and progression (Pan et al., 2024). KRT17 enhances proliferation, migration, and inflammatory cytokine release through activation of mTOR/S6K1 signaling (Li et al., 2020). Collectively, these CRGs are functionally involved in key oncogenic pathways, including metabolism, cell-cycle control, invasion, and immune regulation, consistent with their prognostic relevance in our model.

Our investigation offers a novel mechanistic dimension to the established prognostic signature, particularly regarding the core high-risk gene, KRT17. While classically recognized as a cytoskeletal protein involving in mTOR/S6K1 signaling, our study proposes a “moonlighting” metabolic function for KRT17 through its interaction with the cuproptosis executioner, FDX1 (Tsvetkov et al., 2022). We specifically selected FDX1 for molecular docking because it serves as the upstream gatekeeper of protein lipoylation; its loss is the primary cause of resistance to copper-induced cell death. Our docking data revealed a high-affinity binding interface between KRT17 and FDX1, suggesting that KRT17 may act as a molecular scaffold that sequesters or physically inhibits FDX1. This “sequestration effect” would effectively disarm the cuproptosis machinery, allowing tumor cells to survive even under high copper stress conditions. Furthermore, our single-cell analysis provides critical spatial resolution that resolves the origin of this resistance. By pinpointing KRT17 expression exclusively to malignant epithelial cells—rather than stromal or immune compartments—we demonstrate that the poor prognosis is driven by tumor-intrinsic resistance mechanisms. This aligns with our observation of an immunosuppressive microenvironment (e.g., M2 macrophage enrichment) in high-risk patients; we postulate that KRT17-mediated inhibition of immunogenic cell death (cuproptosis) prevents the release of “danger signals” (DAMPs), thereby potentially fostering an immune-excluded (“cold”) microenvironment. Thus, KRT17 represents a dual-function target: blocking it could potentially reactivate cuproptosis while simultaneously sensitizing “cold” tumors to immunotherapy.

Therapeutic strategies for gastric cancer currently include surgical or endoscopic tumor removal, cytotoxic chemotherapy, targeted agents, and radiotherapy; however, even with incremental progress, many patients continue to experience limited clinical benefit. In recent years, immunotherapy has emerged as a promising approach, in part because it acts directly on the tumor immune microenvironment and is capable of generating more durable antitumor responses (Joshi and Badgwell, 2021; Li et al., 2021). Although immunotherapy offers novel therapeutic promise, a considerable proportion of patients do not experience benefit, primarily due to factors like tumor heterogeneity and the intricate nature of the TIME, areas that necessitate ongoing research (Zeng et al., 2021). The TIME itself is recognized for its profound complexity, comprising elements such as surrounding vasculature, immune cells, fibroblasts, inflammatory cells derived from bone marrow, and ECM. As research has advanced, it has become clear that the tumor microenvironment shapes cancer behavior, and by tipping the immune balance toward suppression rather than cytotoxic activity, it facilitates immune escape and diminishes the efficacy of immunotherapeutic approaches.

Beyond its role as an essential nutrient and signal mediator, metal metabolism has been linked to immune regulation, including antitumor immunity (Wang et al., 2020). Work in this area has highlighted the relevance of copper within the TIME; for example, Voli et al. reported that expression of the copper importer CTR-1 strongly aligned with PD-L1 levels in multiple cancer types, a relationship not observed in normal tissue (Voli et al., 2020). Furthermore, copper supplementation was shown to augment PD-L1 expression at both the mRNA and protein levels in cancer cells via the EGFR and STAT signaling pathways, thereby promoting PD-L1-driven immune evasion in murine models (Chowell et al., 2018). Extensive evidence also confirms the active involvement of mast cells in various pathological conditions, and copper is known to mediate mast cell maturation via MAPK signaling (Hu Frisk et al., 2017). To date, however, the specific function of cuproptosis within the TIME remains unexplored. Significantly, our current study is the inaugural attempt to quantify the relationship between cuproptosis and cancer immunity.

Macrophages constitute the predominant myeloid population in the tumor microenvironment and can acquire either an M1-like or M2-like functional state (Pan et al., 2020). Although capable of exerting tumor-restrictive or tumor-promoting effects, accumulating evidence highlights a strong pro-tumor contribution of M2-biased TAMs in gastric cancer. Zhao and colleagues reported that TAMs isolated from gastric tumors were largely skewed toward the M2 phenotype, and further demonstrated that stromal cells derived from gastric cancer facilitated EMT and metastatic behavior by driving M2 polarization through the IL-6/IL-8–JAK2–STAT3 axis (Li et al., 2019; Wang et al., 2024a). Therapeutic strategies that inhibit M2 macrophage activity have been shown to revive CD8^+^ T-cell function in immunosuppressive settings, and the degree of M2 macrophage infiltration has been linked to 5-year survival outcomes in patients with gastric cancer (Viitala et al., 2019; Junttila et al., 2020). Within our dataset, tumors with higher cuproptosis risk scores exhibited greater infiltration of M2 macrophages, implying that cuproptosis-associated pathways may intersect with macrophage-mediated immune suppression and influence antitumor immunity.

Immunotherapy has become fundamental to gastric cancer management, with checkpoint inhibitors (anti-PD1, anti-CTLA4) continually improving survival and shifting to first-line use. Consequently, identifying reliable biomarkers is crucial. Mismatch repair deficiency (MSI-H) is recognized as an excellent predictor in gastrointestinal tumors. Past studies reported higher MSI-H rates in STAD (19.09% in TCGA, 5.75% in Chinese cohorts) compared to other solid tumors (Bonneville et al., 2017). Clinical trials (KEYNOTE-061, -062) demonstrated that anti-PD1 therapy provided superior OS and ORR versus chemotherapy for MSI-H gastric cancer patients (Shitara et al., 2020; Shitara et al., 2018). In our analysis, MSI-H tumors tended to cluster within the low-risk category, and patients with MSI-H displayed noticeably lower risk scores than those with MSI-L/MSS disease. Moreover, numerous immune checkpoint genes varied significantly between the two cuproptosis risk groups, suggesting that cuproptosis-related biology may intersect with checkpoint regulation and warrants further investigation. Since T cell cytotoxicity depends on effective HLA-mediated antigen presentation, and HLA profiling predicts immunotherapy response (Chowell et al., 2018), lower expression of HLA-DMA and HLA-F in the high-risk group points to their potential relevance for predicting checkpoint response and guiding neoantigen vaccine development. High cuproptosis scores aligned with weaker immunogenic features, including reduced MSI-H frequency, lower checkpoint and TMB levels, and limited effector immune infiltration. These results suggest that cuproptosis may contribute to immune escape in STAD and support further evaluation of cuproptosis-targeted strategies, alone or combined with immunotherapy.

Several constraints of this study should be noted and interpreted with caution. Firstly, prior TCGA-based survival analyses in oral squamous cell carcinoma and bladder cancer have illustrated both the value and the boundaries of such approaches (Wang et al., 2024b). Bulk transcriptomic studies are also prone to technical and biological biases, including sequencing platform variability, tumor heterogeneity, and sample purity, which may confound gene–outcome associations (Liu et al., 2025). Correlation-based findings cannot be directly taken as causal, and discrepancies between mRNA and protein levels may further complicate interpretation. In addition, although our pipeline is consistent with established frameworks for tumor microenvironment–associated prognostic modeling, experimental validation remains essential to confirm computational predictions. Although we provided computational validation via molecular docking and single-cell resolution analysis to strengthen our mechanistic hypothesis, further wet-lab experiments such as Co-IP are warranted to definitively confirm the physical binding between KRT17 and FDX1. Furthermore, while our study highlighted the correlation between the cuproptosis risk signature and the immune landscape (e.g., M2 macrophage polarization and checkpoint expression), we acknowledge that these findings are primarily based on bioinformatic associations and bulk tissue validation. Direct functional validation of tumor–immune interactions using *in vitro* models remains to be performed. Recent pivotal studies have established elegant frameworks for addressing this via co-culture systems. For instance, a recent study employed co-culture assays to demonstrate how tumor-derived signals modulate the immune microenvironment (Guo et al., 2025). Similarly, a research utilized transwell assays to evaluate the impact of specific gene alterations on macrophage M1/M2 polarization and inflammatory cytokine production (Orlandi et al., 2023). Drawing upon these established methodologies, our future work will focus on establishing *in vitro* co-culture systems (e.g., gastric cancer cells with PBMCs or THP-1 derived macrophages) to definitively verify whether KRT17 knockdown directly inhibits M2 macrophage polarization and alters PD-L1 expression, thereby moving from association to causation. Finally, the sample size of our in-house clinical validation cohort (n = 14) for RT-qPCR was relatively small due to the strict time window for sample collection and ethical constraints. We acknowledge that this limited sample size may constrain the statistical power of our experimental validation. However, to compensate for this limitation and rigorously test the generalizability of our findings, we performed additional validation in a large-scale independent external cohort (GSE62254, n = 300). The highly consistent prognostic value observed in this large public dataset supports the reliability and robustness of our 5-gene signature, reinforcing our conclusions despite the limited scale of our primary clinical samples. Notably, we observed a divergence between the mRNA levels (validated by our RT-qPCR) and protein levels (retrieved from the HPA database) for certain risk genes, specifically RPL39L and SYNPO2. While our transcriptomic data provided quantitative evidence of expression changes, HPA staining indicated low or non-detected protein abundance. Such non-linear mRNA-protein concordance is a well-recognized phenomenon in cancer biology (Vogel and Marcotte, 2012). For RPL39L, this inconsistency likely stems from post-transcriptional regulation, where “orphan” ribosomal proteins failing to assemble into ribosomes are subject to rapid ubiquitin-proteasome degradation, reducing protein stability despite high mRNA availability (Sung et al., 2016). Regarding SYNPO2, the discrepancy may be attributed to the existence of multiple isoforms or distinct cellular localization. SYNPO2 undergoes extensive alternative splicing to generate functionally distinct isoforms, and antibodies used in HPA may not recognize the specific tumor-associated variants (Kai and Duncan, 2013). Furthermore, recent studies and our own single-cell analysis suggest SYNPO2 expression correlates with immune infiltration (e.g., macrophages and mast cells); thus, bulk RT-qPCR detects signals from the stroma/immune compartment, whereas IHC scoring in HPA often focuses on malignant cells, potentially leading to a “false negative” interpretation in tumor tissue (Uhl et al., 2015; Ye et al., 2024). Therefore, while HPA data provide a valuable reference, our patient-derived RT-qPCR results offer a more direct and robust assessment of the transcriptional state in our specific cohort. Looking forward, emerging approaches such as single-cell transcriptomics and AI-based methods like generative adversarial networks may help overcome current biases and provide more precise insights.

## 5 Conclusion

To sum up, we established a prognostically meaningful model derived from cuproptosis-related genes in STAD. The study thoroughly investigated the associations between cuproptosis and factors related to tumor immunity. Our findings establish a foundation for exploring innovative, novel approaches encompassing targeted therapy and immunotherapy for STAD. Future studies should verify the clinical predictive value of the cuproptosis score in immunotherapy settings and dissect the mechanisms underlying the association between cuproptosis and immune regulation in GC.

## Data Availability

The original contributions presented in the study are included in the article/Supplementary Material. Further inquiries can be directed to the corresponding authors. The datasets analyzed for this study can be found in the following repositories: 1. The Cancer Genome Atlas (TCGA) database: https://portal.gdc.cancer.gov/ (Project ID: TCGA-STAD). 2. Gene Expression Omnibus (GEO): GSE84433 https://www.ncbi.nlm.nih.gov/geo/query/acc.cgi?acc=GSE84433; GSE62254 (ACRG cohort) https://www.ncbi.nlm.nih.gov/geo/query/acc.cgi?acc=GSE62254; GSE163558 (Single-cell RNA-seq) https://www.ncbi.nlm.nih.gov/geo/query/acc.cgi?acc=GSE163558. The raw data supporting the RT-qPCR validation (clinical samples) and the detailed molecular docking results (top 100 models) are provided in the Supplementary Material.

## References

[B1] Blockhuys S. Celauro E. Hildesjö C. Feizi A. Stål O. Fierro-González J. C. (2017). Defining the human copper proteome and analysis of its expression variation in cancers. Metallomics 9 (2), 112–123. 10.1039/c6mt00202a 27942658

[B2] Bonneville R. Krook M. A. Kautto E. A. Miya J. Wing M. R. Chen H. Z. (2017). Landscape of microsatellite instability across 39 cancer types. JCO Precis. Oncol. 2017. 10.1200/po.17.00073 29850653 PMC5972025

[B3] Chong W. Ren H. Chen H. Xu K. Zhu X. Liu Y. (2024). Clinical features and molecular landscape of cuproptosis signature-related molecular subtype in gastric cancer. Imeta 3 (3), e190. 10.1002/imt2.190 38898987 PMC11183172

[B4] Chowell D. Morris L. G. T. Grigg C. M. Weber J. K. Samstein R. M. Makarov V. (2018). Patient HLA class I genotype influences cancer response to checkpoint blockade immunotherapy. Science 359 (6375), 582–587. 10.1126/science.aao4572 29217585 PMC6057471

[B5] Ge E. J. Bush A. I. Casini A. Cobine P. A. Cross J. R. DeNicola G. M. (2022). Connecting copper and cancer: from transition metal signalling to metalloplasia. Nat. Rev. Cancer 22 (2), 102–113. 10.1038/s41568-021-00417-2 34764459 PMC8810673

[B6] Guo Z. Chen D. Yao L. Sun Y. Li D. Le J. (2025). The molecular mechanism and therapeutic landscape of copper and cuproptosis in cancer. Signal Transduct. Target Ther. 10 (1), 149. 10.1038/s41392-025-02192-0 40341098 PMC12062509

[B7] Gupte A. Mumper R. J. (2009). Elevated copper and oxidative stress in cancer cells as a target for cancer treatment. Cancer Treat. Rev. 35 (1), 32–46. 10.1016/j.ctrv.2008.07.004 18774652

[B8] Hu Frisk J. M. Kjellén L. Kaler S. G. Pejler G. Öhrvik H. (2017). Copper regulates maturation and expression of an MITF:Tryptase axis in mast cells. J. Immunol. 199 (12), 4132–4141. 10.4049/jimmunol.1700786 29127151 PMC5728160

[B9] Joshi S. S. Badgwell B. D. (2021). Current treatment and recent progress in gastric cancer. CA Cancer J. Clin. 71 (3), 264–279. 10.3322/caac.21657 33592120 PMC9927927

[B10] Junttila A. Helminen O. Väyrynen J. P. Ahtiainen M. Kenessey I. Jalkanen S. (2020). Immunophenotype based on inflammatory cells, PD-1/PD-L1 signalling pathway and M2 macrophages predicts survival in gastric cancer. Br. J. Cancer 123 (11), 1625–1632. 10.1038/s41416-020-01053-7 32943749 PMC7687887

[B11] Kai F. Duncan R. (2013). Prostate cancer cell migration induced by myopodin isoforms is associated with formation of morphologically and biochemically distinct actin networks. Faseb J. 27 (12), 5046–5058. 10.1096/fj.13-231571 24005909

[B12] Li W. Zhang X. Wu F. Zhou Y. Bao Z. Li H. (2019). Gastric cancer-derived mesenchymal stromal cells trigger M2 macrophage polarization that promotes metastasis and EMT in gastric cancer. Cell Death Dis. 10 (12), 918. 10.1038/s41419-019-2131-y 31801938 PMC6892854

[B13] Li D. Ni X. F. Tang H. Zhang J. Zheng C. Lin J. (2020). KRT17 functions as a tumor promoter and regulates proliferation, migration and invasion in pancreatic cancer via mTOR/S6k1 pathway. Cancer Manag. Res. 12, 2087–2095. 10.2147/cmar.S243129 32256116 PMC7090205

[B14] Li K. Zhang A. Li X. Zhang H. Zhao L. (2021). Advances in clinical immunotherapy for gastric cancer. Biochim. Biophys. Acta Rev. Cancer 1876 (2), 188615. 10.1016/j.bbcan.2021.188615 34403771

[B15] Liu H. Dilger J. P. (2025). Different strategies for cancer treatment: targeting cancer cells or their neighbors? Chin. J. Cancer Res. 37 (2), 289–292. 10.21147/j.issn.1000-9604.2025.02.12 40353083 PMC12062981

[B16] Liu H. Li Y. Karsidag M. Tu T. Wang P. (2025). Technical and biological biases in bulk transcriptomic data mining for cancer research. J. Cancer 16 (1), 34–43. 10.7150/jca.100922 39744578 PMC11660120

[B17] Orlandi G. Roncucci L. Carnevale G. Sena P. (2023). Different roles of apoptosis and autophagy in the development of human colorectal cancer. Int. J. Mol. Sci. 24 (12), 24. 10.3390/ijms241210201 37373349 PMC10299161

[B18] Pan Y. Yu Y. Wang X. Zhang T. (2020). Tumor-associated macrophages in tumor immunity. Front. Immunol. 11, 583084. 10.3389/fimmu.2020.583084 33365025 PMC7751482

[B19] Pan C. Dai J. Wei Y. Yang L. Ding Z. Wang X. (2024). Matrix metalloproteinase 11 promotes migration and invasion of colorectal cancer by elevating slug protein. Int. J. Med. Sci. 21 (11), 2170–2188. 10.7150/ijms.98007 39239548 PMC11373555

[B20] Seeneevassen L. Bessède E. Mégraud F. Lehours P. Dubus P. Varon C. (2021). Gastric cancer: advances in carcinogenesis research and new therapeutic strategies. Int. J. Mol. Sci. 22 (7), 3418. 10.3390/ijms22073418 33810350 PMC8037554

[B21] Shanbhag V. Jasmer-McDonald K. Zhu S. Martin A. L. Gudekar N. Khan A. (2019). ATP7A delivers copper to the lysyl oxidase family of enzymes and promotes tumorigenesis and metastasis. Proc. Natl. Acad. Sci. U. S. A. 116 (14), 6836–6841. 10.1073/pnas.1817473116 30890638 PMC6452744

[B22] Shitara K. Özgüroğlu M. Bang Y. J. Di Bartolomeo M. Mandalà M. Ryu M. H. (2018). Pembrolizumab versus paclitaxel for previously treated, advanced gastric or gastro-oesophageal junction cancer (KEYNOTE-061): a randomised, open-label, controlled, phase 3 trial. Lancet 392 (10142), 123–133. 10.1016/s0140-6736(18)31257-1 29880231

[B23] Shitara K. Van Cutsem E. Bang Y. J. Fuchs C. Wyrwicz L. Lee K. W. (2020). Efficacy and safety of pembrolizumab or pembrolizumab plus chemotherapy vs chemotherapy alone for patients with first-line, advanced gastric cancer: the KEYNOTE-062 phase 3 randomized clinical trial. JAMA Oncol. 6 (10), 1571–1580. 10.1001/jamaoncol.2020.3370 32880601 PMC7489405

[B24] Sung M. K. Porras-Yakushi T. R. Reitsma J. M. Huber F. M. Sweredoski M. J. Hoelz A. (2016). A conserved quality-control pathway that mediates degradation of unassembled ribosomal proteins. Elife 5. 10.7554/eLife.19105 27552055 PMC5026473

[B25] Sung H. Ferlay J. Siegel R. L. Laversanne M. Soerjomataram I. Jemal A. (2021). Global cancer statistics 2020: GLOBOCAN estimates of incidence and mortality worldwide for 36 cancers in 185 countries. CA Cancer J. Clin. 71 (3), 209–249. 10.3322/caac.21660 33538338

[B26] Tsvetkov P. Coy S. Petrova B. Dreishpoon M. Verma A. Abdusamad M. (2022). Copper induces cell death by targeting lipoylated TCA cycle proteins. Science 375 (6586), 1254–1261. 10.1126/science.abf0529 35298263 PMC9273333

[B27] Uhlén M. Fagerberg L. Hallström B. M. Lindskog C. Oksvold P. Mardinoglu A. (2015). Proteomics. Tissue-based map of the human proteome. Science 347 (6220), 1260419. 10.1126/science.1260419 25613900

[B28] Viitala M. Virtakoivu R. Tadayon S. Rannikko J. Jalkanen S. Hollmén M. (2019). Immunotherapeutic blockade of macrophage Clever-1 reactivates the CD8(+) T-cell response against immunosuppressive tumors. Clin. Cancer Res. 25 (11), 3289–3303. 10.1158/1078-0432.Ccr-18-3016 30755440

[B29] Vogel C. Marcotte E. M. (2012). Insights into the regulation of protein abundance from proteomic and transcriptomic analyses. Nat. Rev. Genet. 13 (4), 227–232. 10.1038/nrg3185 22411467 PMC3654667

[B30] Voli F. Valli E. Lerra L. Kimpton K. Saletta F. Giorgi F. M. (2020). Intratumoral copper modulates PD-L1 expression and influences tumor immune evasion. Cancer Res. 80 (19), 4129–4144. 10.1158/0008-5472.Can-20-0471 32816860

[B31] Wang C. Zhang R. Wei X. Lv M. Jiang Z. (2020). Metalloimmunology: the metal ion-controlled immunity. Adv. Immunol. 145, 187–241. 10.1016/bs.ai.2019.11.007 32081198

[B32] Wang Y. Zhang L. Zhou F. (2022). Cuproptosis: a new form of programmed cell death. Cell Mol. Immunol. 19 (8), 867–868. 10.1038/s41423-022-00866-1 35459854 PMC9338229

[B33] Wang Y. Wang Z. Li S. Ma J. Dai X. Lu J. (2024a). Deciphering JAK/STAT signaling pathway: a multifaceted approach to tumorigenesis, progression and therapeutic interventions. Int. Immunopharmacol. 131, 111846. 10.1016/j.intimp.2024.111846 38520787

[B34] Wang Y. Wang J. Zeng T. Qi J. (2024b). Data-mining-based biomarker evaluation and experimental validation of SHTN1 for bladder cancer. Cancer Genet. 288-289, 43–53. 10.1016/j.cancergen.2024.09.002 39260052

[B35] Wong Q. W. Li J. Ng S. R. Lim S. G. Yang H. Vardy L. A. (2014). RPL39L is an example of a recently evolved ribosomal protein paralog that shows highly specific tissue expression patterns and is upregulated in ESCs and HCC tumors. RNA Biol. 11 (1), 33–41. 10.4161/rna.27427 24452241 PMC3929422

[B36] Ye G. Tu L. Li Z. Li X. Zheng X. Song Y. (2024). SYNPO2 promotes the development of BLCA by upregulating the infiltration of resting mast cells and increasing the resistance to immunotherapy. Oncol. Rep. 51 (1), 14. 10.3892/or.2023.8673 38038167 PMC10758676

[B37] Zeng D. Wu J. Luo H. Li Y. Xiao J. Peng J. (2021). Tumor microenvironment evaluation promotes precise checkpoint immunotherapy of advanced gastric cancer. J. Immunother. Cancer 9 (8), e002467. 10.1136/jitc-2021-002467 34376552 PMC8356190

[B38] Zheng Z. Song Y. (2023). Synaptopodin-2: a potential tumor suppressor. Cancer Cell Int. 23 (1), 158. 10.1186/s12935-023-03013-6 37544991 PMC10405370

