# Supplementary material for: Cuproptosis-related gene signatures define immune subtypes and predict prognosis in gastric cancer
Source: Front Mol Biosci. 2026 Feb 9;13:1746613. doi: 10.3389/fmolb.2026.1746613 (PMC12926107; doi:10.3389/fmolb.2026.1746613)
Supplement: Supplementary file 1 [file DataSheet1.pdf]

Supplementary Figures

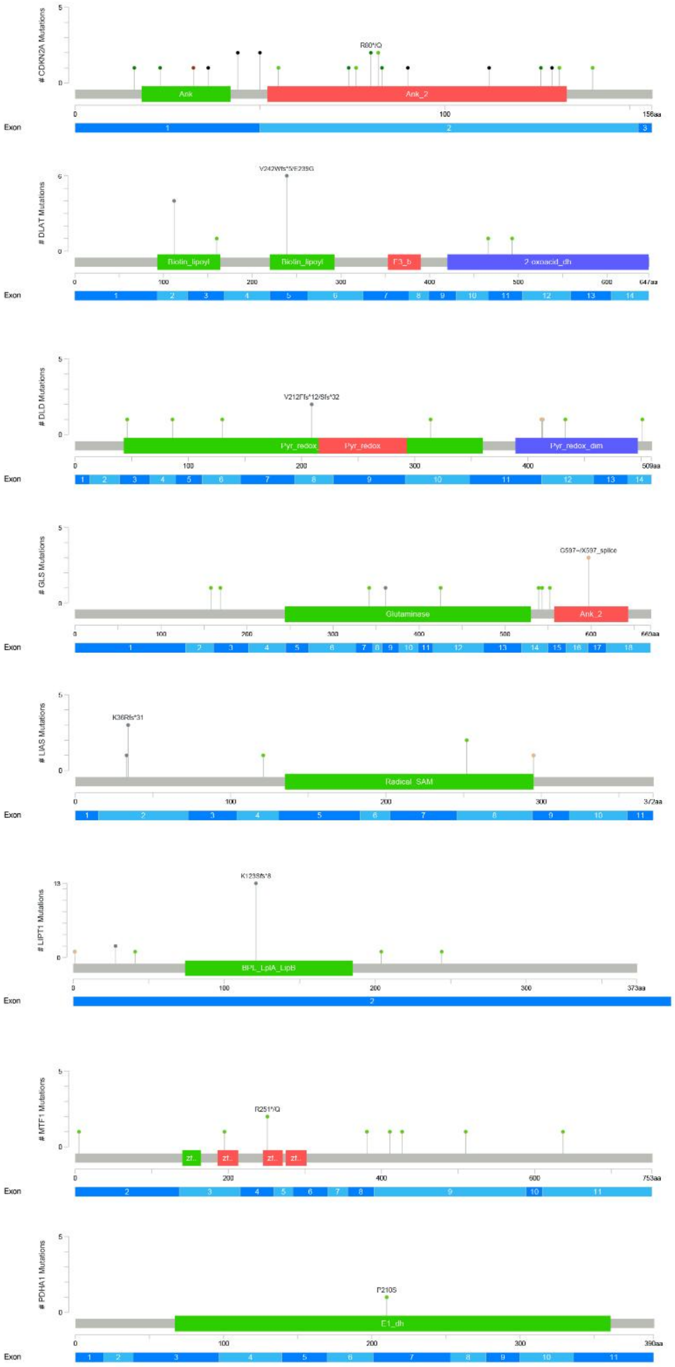

Fig S1: Mutation site analysis of cuproptosis genes according to Cbioportal database.

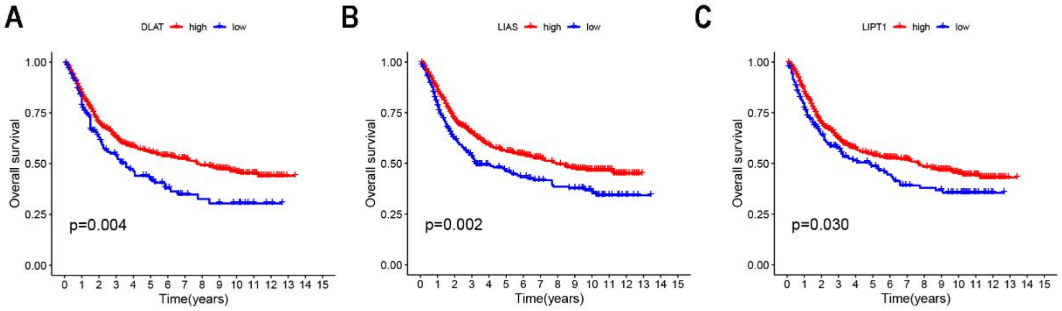

Figure S2: Kaplain-Meier analysis based on expression of cuproptosis genes, including *DLAT, LIAS* and *LIPT1*.

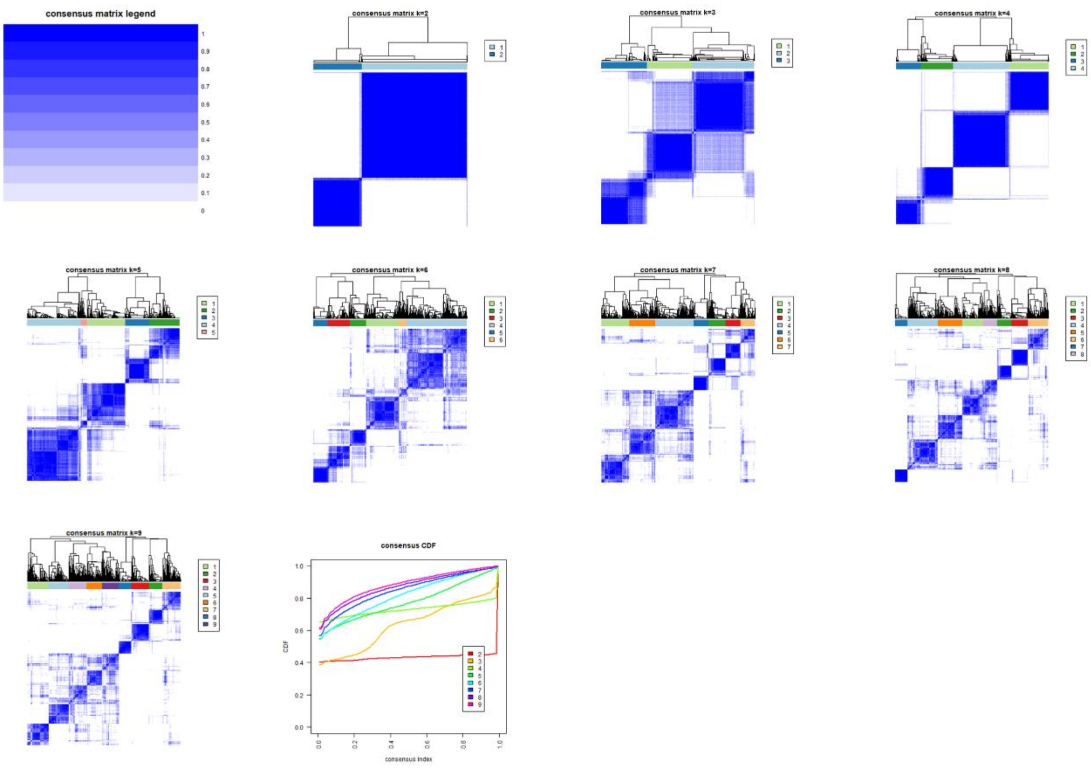

Figure S3: Unsupervised clustering of cuproptosis genes and Consensus matrix heatmaps for k = 1-9.

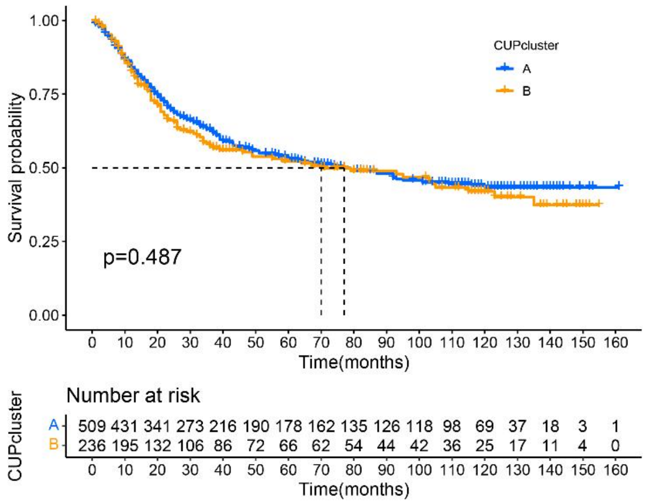

Figure S4: Survival analysis of cuproptosis cluster according to OS in STAD.

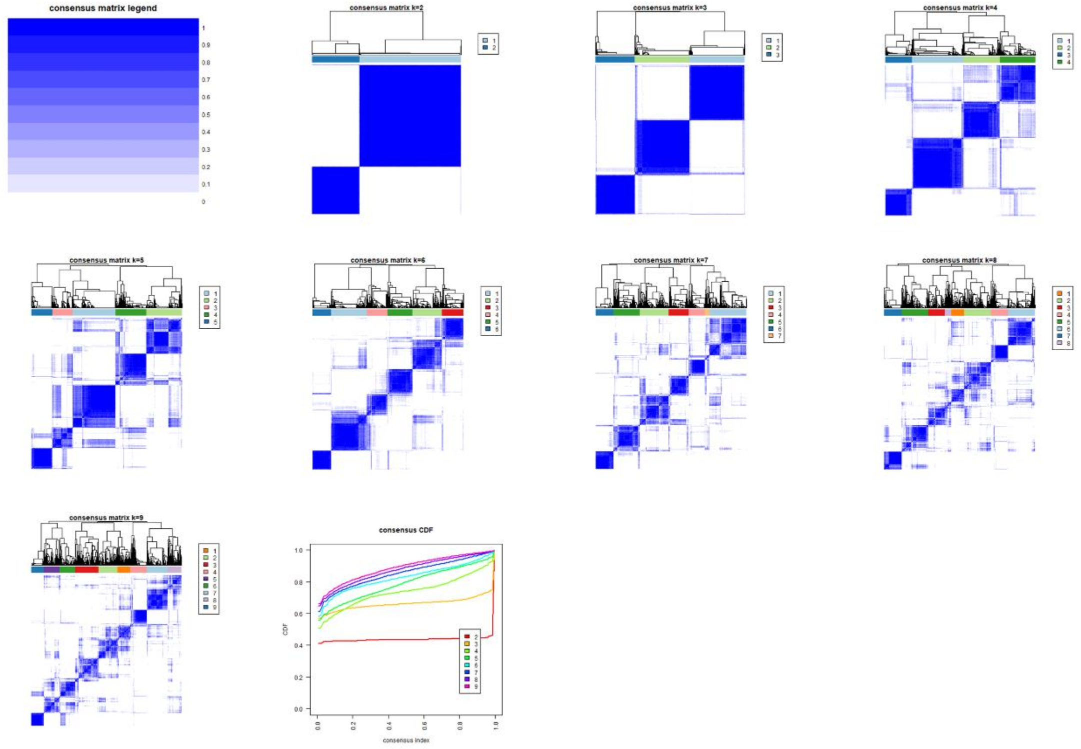

Figure S5: Identification of cuproptosis gene cluster in STAD samples and consensus matrix heatmaps for k = 1-9.

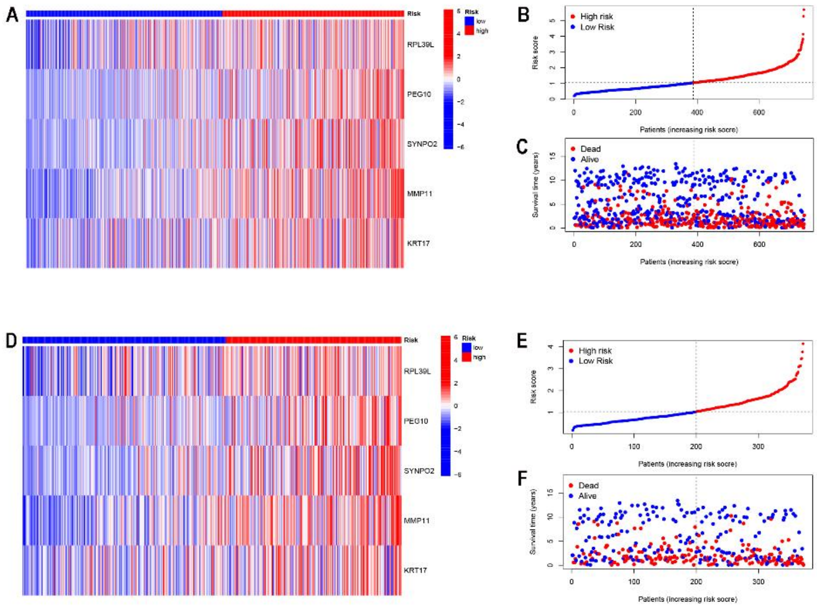

Figure S6: Genes expression in the cuproptosis risk score between the high and low-risk groups. Cuproptosis risk score distribution survival status of STAD patients. (A, B, C in the all patients set and D, E, F in the validating set)

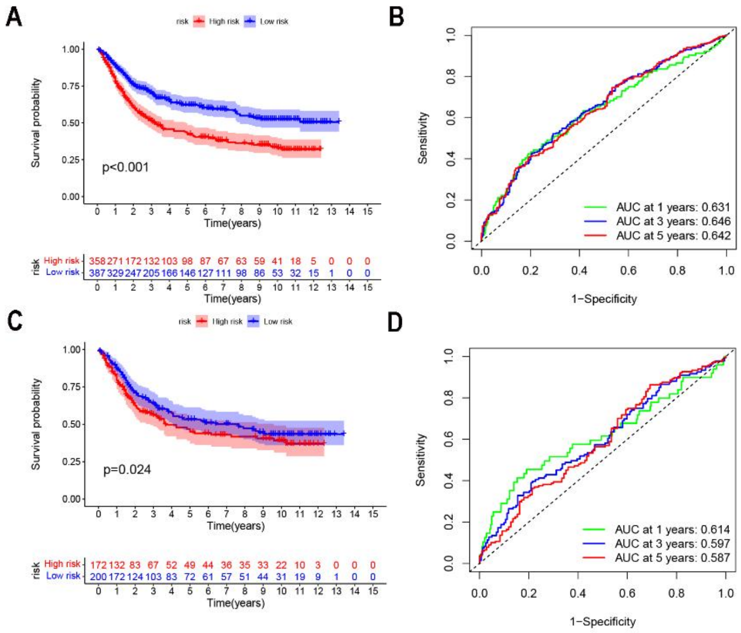

Figure S7: Overall survival curves and ROC curves of risk group in the all patients set (A, B) and validating set (C, D).

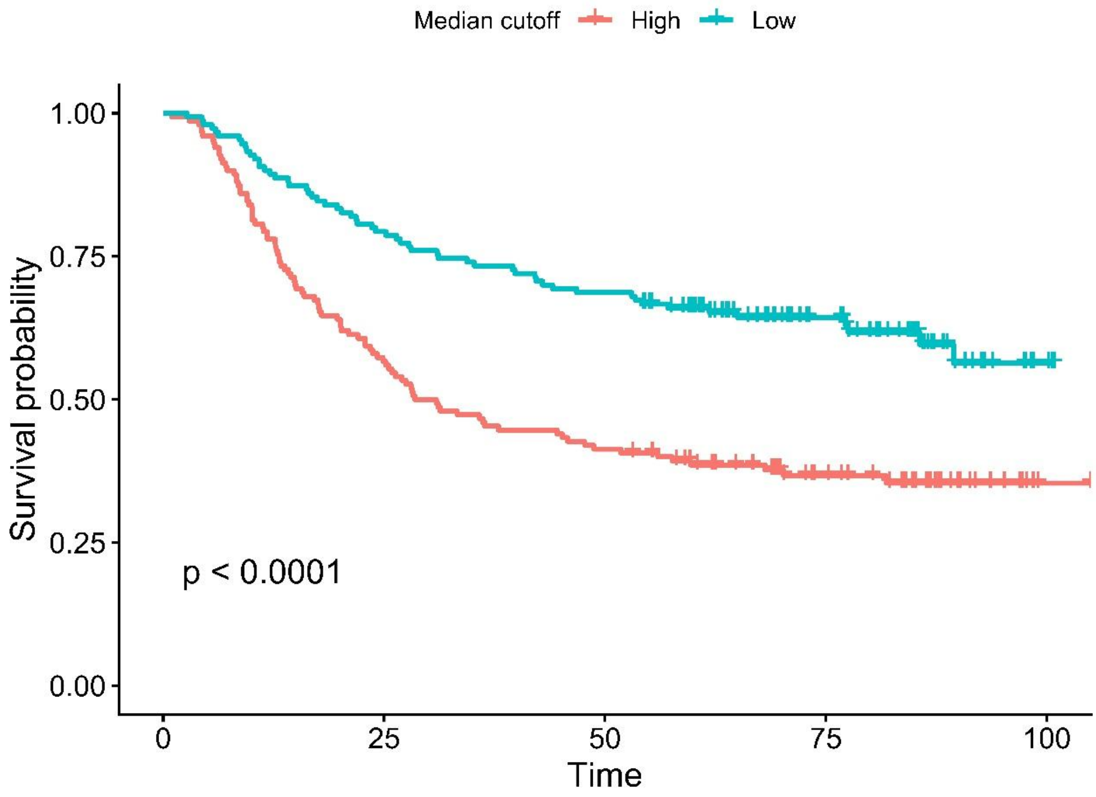

Fig.S8: Validation of the prognostic reliability of the 5-gene cuproptosis signature in an independent external cohort.

Kaplan-Meier survival analysis of overall survival (OS) in the GSE62254 (ACRG) cohort (n=300). Patients were stratified into high-risk (red line) and low-risk (blue line) groups based on the median risk score calculated by the 5-gene signature. The high-risk group exhibited significantly poorer prognosis compared to the low-risk group ($p < 0.0001$, Log-rank test).

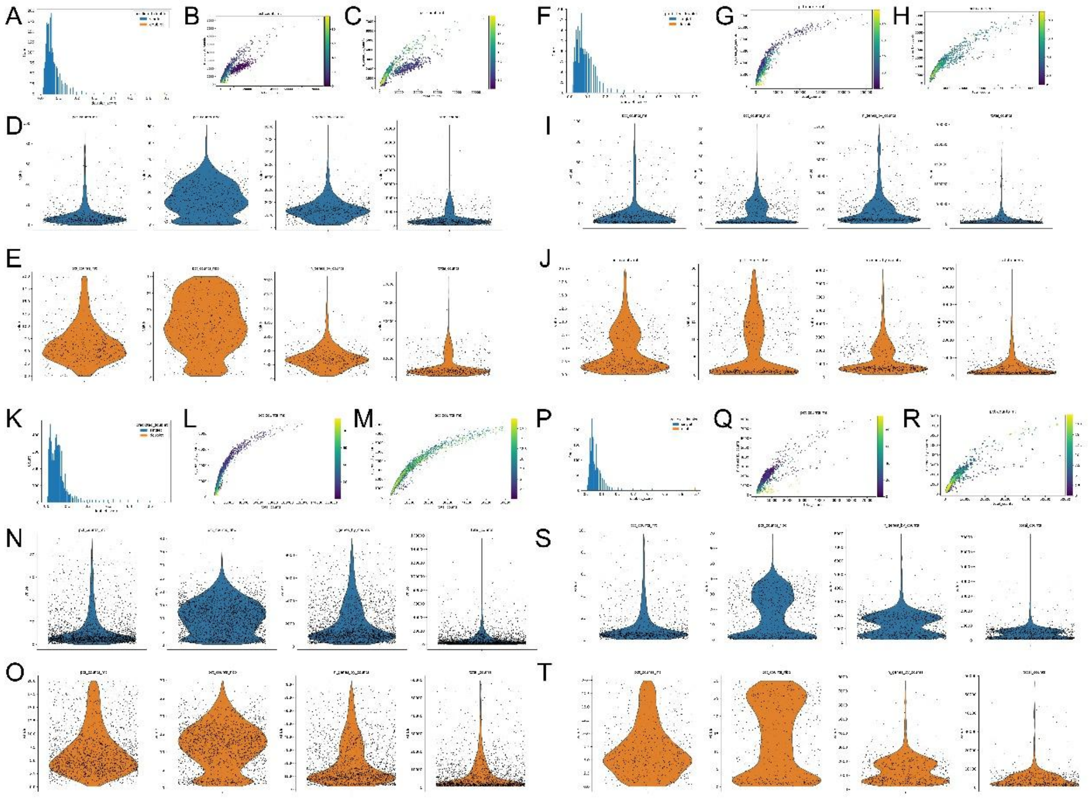

FigureS9. Quality control metrics for single-cell RNA sequencing data.
Violin plots and scatter plots displaying the distribution of quality control metrics across the analyzed samples (including normal tissue NT1 and tumor tissues PT1, PT2, PT3). The visualization includes the number of detected genes (nFeature_RNA), sequencing depth (nCount_RNA), and the percentage of mitochondrial gene expression (percent.mt). These metrics were utilized to filter out low-quality cells and remove potential doublets prior to downstream dimensionality reduction and clustering.

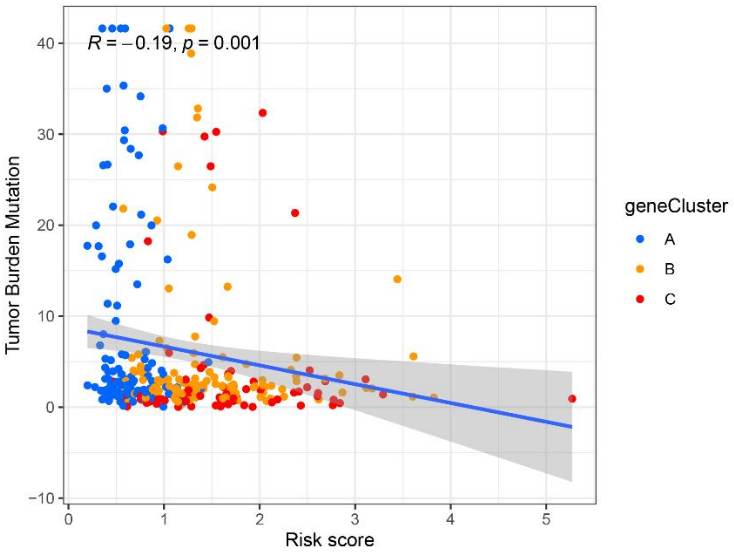

Figure S10: Correlation of TMB score with cuproptosis gene clusters.
